# Future horizons in the analysis of technical-tactical performance in women’s football: a mixed methods approach to the analysis of in-depth interviews with professional coaches and players

**DOI:** 10.3389/fpsyg.2023.1128549

**Published:** 2023-05-18

**Authors:** Iyán Iván-Baragaño, Antonio Ardá, M. Teresa Anguera, José Luis Losada, Rubén Maneiro

**Affiliations:** ^1^Faculty of Sport Sciences, Universidad Europea de Madrid, Madrid, Spain; ^2^Department of Physical and Sport Education, University of A Coruña, A Coruña, Spain; ^3^Faculty of Psychology, Institute of Neurosciences, University of Barcelona, Barcelona, Spain; ^4^Department of Social Psychology and Quantitative Psychology, University of Barcelona, Barcelona, Spain; ^5^Department of Science of Physical Activity and Sport, Pontifical University of Salamanca, Salamanca, Spain

**Keywords:** women’s football, observational methodology, performance indicators, in-depth interviews, indirect observation, quantitizing

## Abstract

**Introduction:**

Scientific knowledge about the criteria that determine success in women’s football is beginning to develop.

**Methods:**

This study was carried out with the aim of detecting regularities in the offensive success in elite women’s football, as well as carrying out an interrelational analysis of linked behaviors, based on in-depth interviews with professional coaches and players. Eight in-depth interviews were conducted with professional Spanish coaches and players. The interviews were analyzed by indirect observation from a process of “quantitizing,” through the construction of an indirect observation *ad hoc* instrument. The segmentation of the transcription of the interviews was carried out in textual units, and the creation of a matrix of codes. Two types of analysis were performed: first, a lag sequential analysis (LSA) was performed and, then, a polar coordinates analysis (PCA), which allowed to find, respectively, a wide number of established communicative patterns with offensive performance in women’s football, as well as an interrelational map between the established codes.

**Results:**

The results obtained allowed us to suggest a statistically significant association between success in women’s football and criteria such as the physical characteristics of a particular player, the individual action space, the duration of the attack, the type of dynamic start, individual and collective technical and tactical aspects, decision making and the type of attack used.

**Discussion:**

Based on these results, the influence of these criteria on performance in women’s soccer can be studied in future studies. In addition, with the aim of increasing the validity of these conclusions, new studies on this subject may be carried out following strategies such as the Delphi Method.

## 1. Introduction

Women’s football has significantly increased its media impact in recent years. The last FIFA Women’s World Cup was seen by 1.12 billion spectators, 106% more than the previous edition (FIFA, 2019a). Currently, approximately 30 million players play football worldwide (FIFA, 2019b). Despite this growth, women’s football is in an inferior position compared to its male counterpart (Lago et al., 2021).

In the scientific field, the first article on women’s football was published in 1939 (Okholm Kryger et al., 2021). Despite this, the first year in which more than 10 articles on this topic were published was 1997 (Okholm Kryger et al., 2021). Currently, the number of scientific publications on women’s football represents approximately a quarter of the total on this sport (Kirkendall, 2020). In addition, most studies have focused on the anatomical and physiological aspects related to the physical performance of the players (Kirkendall and Krustrup, 2021).

With the aim of enhancing the visibility and participation of women footballers, new strategies are needed to promote this sport. In this regard, recent research showed that the performance of women’s football teams was higher compared to men’s teams in those countries with a higher degree of women’s empowerment, better domestic leagues, and a greater number of players (Lago et al., 2021). This study, on the other hand, showed that the GDP *per capita*, the number of men and women on the executive committees, or the existence of a strategy for women’s football, were variables that did not modify the difference in performance between male and female teams in the same country in the FIFA Ranking (Lago et al., 2021). Therefore, the competent authorities in the field of sport must focus their efforts in the coming years on two fundamental aspects: (i) increasing the number of women who play football and (ii) improving the sports performance of domestic leagues.

Sports performance in team sports, and football in particular, can only be understood from a multifactorial approach (psychological, sociological, physiological, technical-tactical,…), product of the dynamic interaction between competitors through play actions (Preciado et al., 2019). In relation to technical and tactical performance, research in men’s football has provided numerous pieces of evidence in the last two decades (Lago, 2009; Tenga et al., 2010; Lago-Ballesteros et al., 2012; Casal et al., 2017; Sarmento et al., 2018). On the other hand, although knowledge about women’s football, from areas such as physiology and medicine has been developing in recent decades (Okholm Kryger et al., 2021), according to Kirkendall and Krustrup (2021) there are only 21 articles published with the topic match analysis, 19 of which have been published in the last decade and 40 articles (35 in the last 10 years) with the topic match performance. This is why it is an issue that must be developed in the near future.

On this subject, some recent studies have tried to provide evidence when it comes to understanding which are the criteria that determine a higher performance during a match in women’s football. The influence of the match status criterion on the collective behavior in women’s football has recently been demonstrated in set pieces (Lee and Mills, 2021) and ball possessions (Maneiro et al., 2020). Other studies have tried to find out which variables determine the outcome of games in women’s football. In relation to this issue, De Jong et al. (2020) observed that the variable that most influenced the outcome of the match was scoring first. For their part, Kubayi and Larkin (2020) found technical differences between the winning and losing teams in the last FIFA Women’s World Cup France 2019: the teams that won their matches made more passes, shots, and shots on target per game, in addition to having a higher percentage of ball possession and air duels won, this last variable also demonstrated by De Jong et al. (2020). Tactical differences between winning and losing teams were also found in FIFA Women’s World Cup France 2019 (Iván-Baragaño et al., 2022). These authors showed that the teams that won their matches were able to keep possession of the ball for longer on the rival field, as well as to develop a greater number of possessions in the first minutes of the match, characterized by a greater dispute over possession of the ball. On the other hand, an analysis of the FIFA Women’s World Cup Canada 2015 showed that the type of start was a criterion that significantly influenced the creation of scoring opportunities (Scanlan et al., 2020), an aspect that agrees with the findings obtained in the FIFA Women’s World Cup 2019 (Iván-Baragaño et al., 2021). This last study, in turn, demonstrated a probability of offensive success in ball possessions of 75.2% based on the criteria zone of possession, initial offensive intention, and starting zone (Iván-Baragaño et al., 2021), agreeing with the results obtained by Maneiro et al. (2021) 4 years earlier: these authors demonstrated that the result of ball possessions was significantly conditioned by temporality, initial offensive intention or the number of passes, among other criteria. Technical differences have also been found in the development of the game based on the surface of the playing field (García-Unanue et al., 2020).

Despite the recent growth of scientific knowledge, the available evidence is still scarce. For this reason, it is not possible to know if everything we know about men’s football performance can be applied to play in women, taking into account the differences in play between the two sexes (Bradley et al., 2014; Casal et al., 2021; Garnica-Caparrós and Memmert, 2021). Answering this question is considered necessary since it can allow professionals in this sport to apply the knowledge acquired about men’s football to their own sports specialty (Okholm Kryger et al., 2021). Based on the above, it can be said that we are still facing a study area with short-term growth potential. Researchers will undoubtedly have to further develop this issue in the coming years. In this sense, the experience and knowledge of female football coaches and players can help guide research problems, and conducting interviews with the real participants and connoisseurs of the sport can be an effective tool, as demonstrated in other areas of knowledge (García-Fariña et al., 2018, 2021; Del Giacco et al., 2020; Alvarado et al., 2021; Nunes et al., 2022). In the coming years, clubs, public bodies, and researchers must direct their efforts toward achieving the following objectives (Nassis et al., 2021): (i) to know the needs of female football coaches and players, (ii) to intensify scientific production on female football, (iii) through the integration of coaches and players in the studies carried out, and (iv) in search of an improvement in sports practice and the performance of female football teams.

For all the above reasons, this study was carried out with the aim of detecting regularities in the offensive success in elite women’s football, as well as carrying out an interrelational analysis of linked behaviors, based on in-depth interviews with professional coaches and players. Under this premise, we have tried to integrate the practical knowledge, based on the experience in the field of play, of coaches and players of women’s football from the realization of in-depth interviews, together with the theoretical knowledge of experienced researchers in women’s football. On the basis of the analysis of the answers and the conclusions that can be obtained from this study, we hoped to be able to orient the accomplishment of future studies that can help to increase the performance of teams and selections in female football.

## 2. Materials and methods

### 2.1. Design

To carry out this study, the observational methodology was applied, based on the transcription of in-depth interviews carried out with coaches and players, due to their suitability in the analysis and observation of human behavior in natural and spontaneous contexts (Anguera, 1979). It was a nomothetic design -various units of study-, punctual -a single session, although with intra-sessional follow-up-, and multidimensional -various dimensions, as can be seen in the observation instrument- (Anguera et al., 2011).

Due to the nature of the textual material obtained from audio transcriptions of the verbal behavior of coaches and players, it was necessary to conduct the study through indirect observation (Anguera et al., 2018), which is a modality of observational methodology that focuses on situations where perceptivity is limited (there is no visual perception), and the data was obtained from various sources, mostly of a textual nature. Through liquifying (Anguera, 2020), and from the perspective of mixed methods, it is possible to quantitatively analyze qualitative material (in our study, transcriptions of in-depth interviews), which is perfectly compatible with the logic of observational methodology (Anguera et al., 2020). The use of indirect observation requires overcoming challenges derived from partial perceptivity, which have been attempted to be addressed in this research. We highlight the contribution that it provides in studies in which human communication is of interest as a source of information (García-Fariña et al., 2018, 2021; Del Giacco et al., 2020; Alvarado et al., 2021; Nunes et al., 2022).

### 2.2. Participants and sample

A total of 8 in-depth interviews were conducted with professional female football coaches and players. The choice of coaches and players was based on the need to obtain information, through personal stories, on a wide and varied professional background (Solstad et al., 2021). Due to the intensive nature (obtaining many cases from each case) of the indirect observation studies, this number of interviews was considered sufficient for this study, as were others (Sarmento et al., 2014, 2020; Iván-Baragaño et al., 2021). The transcript obtained from the interviews conducted consisted of 73,046 words and 2,410 textual units.

The coaches and players interviewed were selected by expert sampling provided that they met the following inclusion criteria: (i) for coaches: being in possession of the UEFA Pro title as a coach and having at least 1 year of experience as a coach in the Spanish First Division and, (ii) for players: having been called by the Spanish women’s team and having at least 3 years of experience in the Spanish First Division.

A total of 5 coaches (4 men; 1 woman; 39.2 ± 7.85 years) and 3 players (26.66 ± 5.68 years) were interviewed. Among the coaches, there was the second coach of the Spanish Women’s Team, a participant in the FIFA Women’s World Cup France 2019, a champion of the Queen’s Cup in 2019, a Doctor of Sports Sciences, and a Graduate in Physical Activity and Sport Sciences. Of the 3 players interviewed, two of them were UEFA Women’s Champions League champions with their clubs, and one of them was selected by her team to play in the FIFA Women’s World Cup France 2019.

### 2.3. Instruments

#### 2.3.1. Semi-structured interviews

The degree of structuring of the interview allowed for obtaining a valid source of information on the participant’s experience (Jackman et al., 2022). Prior to the preparation of the interviews, 5 pilot interviews were conducted with Spanish professional football players. The final in-depth interview consisted of 15 questions and sub-questions and was prepared by an expert committee. Of its three members, two of them had a PhD in Sports Sciences and more than 30 years of experience as researchers in the field between the two, and one of them was a PhD student in Sports Sciences. In addition, the members had a UEFA A or higher qualification at the time of this study.

The interviews conducted were conducted face-to-face, individually, and in an undirected manner. The interviews were conducted in a pre-agreed location near the interviewee’s residence. Three interviews were conducted by videoconference, a method currently accepted in this type of study (Edwards et al., 2021). All of them were directed by one of the authors of the study and recorded in audio. The interviews lasted between 36 and 75 min and were transcribed *ad verbatim*.

#### 2.3.2. Indirect observation instrument

The *ad hoc* instrument of indirect observation used in this study was constructed by combining the ascending path (of the responses to the theoretical framework/regulation) and descending path (vice versa) from the responses of the people interviewed. A progressive process of adaptation and specification of the dimensions and sub-dimensions that make up this instrument has been followed.

The final instrument was a combination of field format and category systems since it allowed for an ideal solution to the high complexity of the situation under study (Caprara and Anguera, 2019). It was composed of two dimensions and 28 sub-dimensions. Catalogs of behavior were developed based on certain sub-dimensions, depending on their content and characteristics. These catalogs are open lists that meet the condition of mutual exclusivity. In contrast, category systems were constructed based on other sub-dimensions, supported by theoretical frameworks and fulfilling the requirements of exhaustiveness and mutual exclusivity. For each of the categories and behaviors of the catalogs of the observation instrument, a definition was developed and examples and counterexamples were provided using the answers of the people interviewed. The indirect observation instrument used can be found in Table 1.

**Table 1:** Indirect observation instrument *ad hoc*.

Dimensions	Subdimensions	Categories and behaviors	
Dimension 1. Initial assessment of the question	D11 Degree of agreement	D111 Affirmation or degree of positive agreement	
		D112 Affirmation or degree of positive agreement justified	
		D113 Denial or degree of negative agreement	
		D114 Denial or degree of negative agreement justified	
		D115 Neutral	
		D116 Neutral justified	
	D12 Emotional assessment	D121 Emotional interpretation	
Dimension 2. Justification of the reply	D21 Gender allusions	D211 Refers to women’s gender or women’s football	
		D212 Refers to male gender or male football	
	D22 Justification based on team, specific position or player	D221 Refers to a team or a set of teams	
		D222 Refers to a specific position	
		D223 Refers to a specific player	
	D23 Justification for the offensive phase	D231 Refers to a quick attack: counterattack or direct attack	
		D232 Refers to a combinative attack or position play	
		D233 Refers to a combination of offensive game models	
	D24 Justification for the system of play	D241 Refers to the system of play	
	D25 Justification for the defensive phase	D251 Refers to the type of marking in the defensive phase	
		D252 Refers to the positioning of a team in the defensive phase	
	D26 Justification for transition	D261 Refers to the offensive transition	
		D262 Refers to the defensive transition	
	D27 Justification for set piece	D271 Refers to set piece actions	
	D28 Justification based on tactical intent	D281 Refers to a team’s offensive tactical intent	
		D282 Refers to the defensive tactical intent of a team	
	D29 Technical justification	D291 Offensive technique	D2911 Individual offensive technique
			D2912 Collective offensive technique
		D292 Defensive technique	
	D210 Tactical justification	D2101 Offensive tactic	D21011 Individual offensive tactic
			D21012 Collective offensive tactic
		D2102 Defensive tactic	D21021 Individual defensive tactic
			D21022 Collective defensive tactic
	D211 Strategic justification	D2111 Refers to the strategic plan during a match or a moment of this	
	D212 Justification based on final result	D2121 Refers to the final result of the match (successful/unsuccessful)	
		D2122 Refers to the final result in a championship, league… (best/worst teams)	
		D2123 Refer to the level difference between teams	
		D2124 Refers to an even level between teams	
	D213 Justification based on match status	D2131 Refers to winning	
		D2132 Refers to losing	
		D2133 Refers to tying	
	D214 Spatial justification	D2141 Refers to zoned regulatory space	
		D2142 Refers to effective play space	
		D2143 Refers to the spatial context of interaction	
		D2144 Refers to action space	
	D215 Temporary justification	D2151 Refers to the temporality of the action	
		D2152 Refers to possession time	
		D2153 Refers to the duration of the attack	
		D2154 Refers to action time	
	D216 Physical/conditional justification	D2161 Refers to the physical characteristics of a particular player	
		D2162 Refers to the physical characteristics of a specific position or line	
		D2163 Refers to the physical characteristics of a team, set of them or generically to women’s or men’s football	
	D217 Psychological justification	D2171 Refers to psychological aspects	
	D218 Justification alluding to the type of start	D2181 Refers to a static startup type	
		D2182 Refers to a dynamic startup type	
	D219 Justification defensive organization	D2191 Refers to an organized or structured defensive structuring	
		D2192 Refers to a disorganized or circumstantial defensive structuring	
	D220 Decision making	D2201 Refers to decision making and perception	
		D2202 Refers to the perception of the match	
	D221 Justification based on the number of players	D2211 Refers to the number of players involved in an action	
	D222 Justification based on number of passes and speed	D2221 Refers to the number of passes that are made in an action	
		D2222 Refers to the speed of play (touches per player)	
	D223 Justification based on knowledge or opinions	D2231 Justifies the answer based on your experience and/or observations as a player or coach	
		D2232 Justifies the answer based on your technical-scientific knowledge	
		D2233 Refers to what they would like based on their opinion	
	D224 Regulatory justification	D2241 Refers to the regulation	
		D2242 Refers to competition format	
	D225 Quantitative/qualitative justification of superiority or inferiority	D2251 Refers to a superiority of the attacking team	
		D2252 Refers to an inferiority of the attacking team	
		D2253 Refers to an equality between teams of qualitative, quantitative or positional types	
	D226 Comparisons	D2261 Makes comparisons (differentiating) between men’s football and women’s football	
		D2262 Makes comparisons (establishing similarities) between men’s football and women’s football	
		D2263 Makes comparisons between competitions, leagues or categories (temporarily simultaneous)	
		D2264 Makes comparisons between different historical moments of women’s football	
	D227 Other justifications	D2271 Refers to the team that belongs to the coach or player (or to any that have belonged)	
		D2272 Refers to football as a collective sport, of common space and cooperation-opposition	
		D2273 Refers to other factors without defining what they are	
		D2274 Refers to the training of players, technicians or training	
	D228 Justification based on the success or outcome of an action	D2281 Linking success in offensive action or in the game in a positive way	
		D2282 Linking to success in an offensive action or in the game in a negative way	
		D2283 Linking success in offensive action or in the game in a neutral way	

### 2.4. Recording instrument

**Figure 1:**
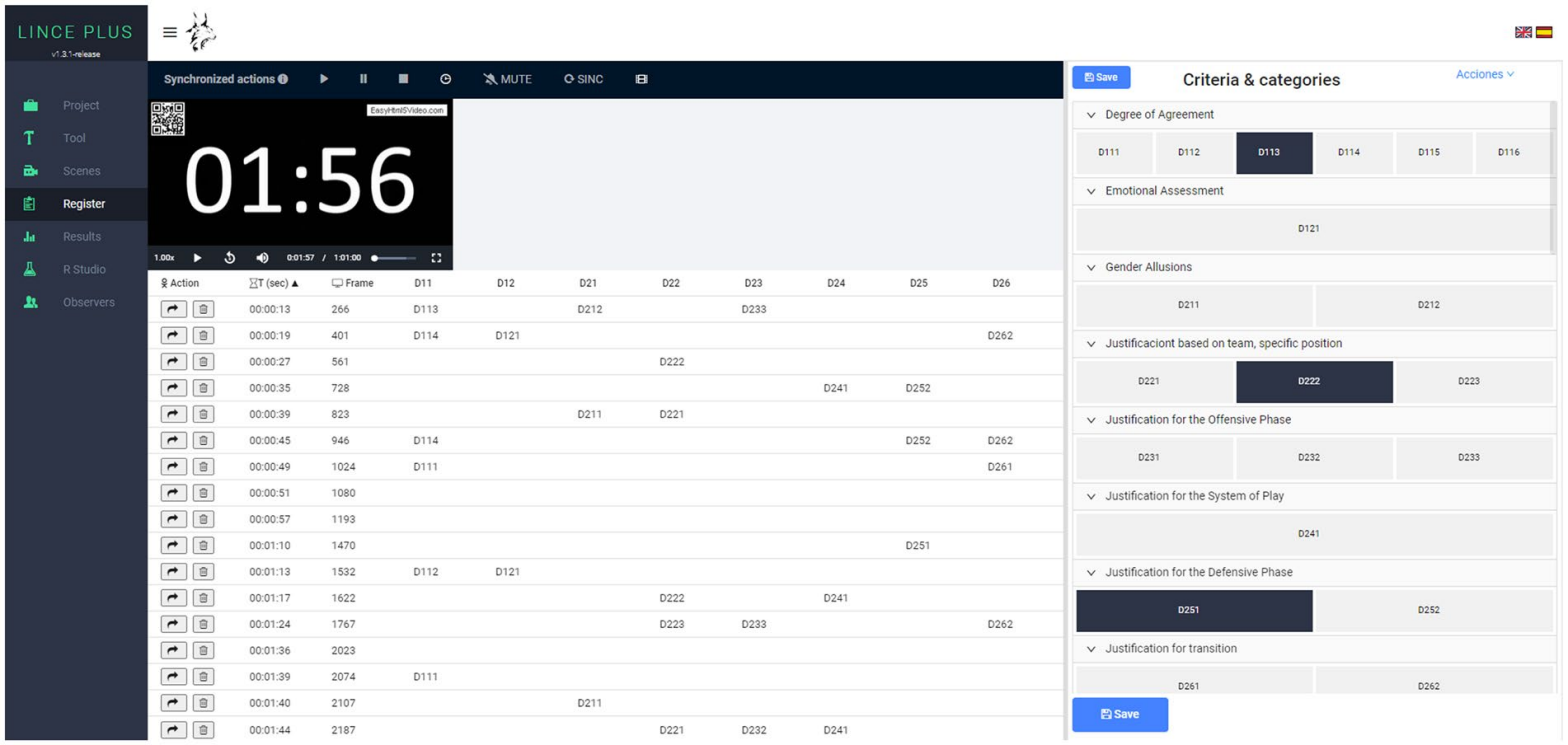
Lince Plus software used in this study. Reproduced with permission from Soto-Fernández et al. (2021).

For the recording and coding of the textual units analyzed, the free software Lince Plus^1^ (Soto-Fernández et al., 2021) of great applicability in observational methodology was used (Figure 1).

As our study involved indirect observation, the successive textual units corresponding to each question’s responses constituted the respective observation units. These units were diachronically recorded as successive rows (formed by one or several codes, depending on the involved sub-dimensions) in the code matrix, which will undergo subsequent analysis.

### 2.5. Procedure

The interviewees were previously informed of the study carried out and agreed to participate in it. This study was approved by the Ethics Committee of the University of A Coruña (Approval code: CEID-UDC-2019-0024).

Due to the increased risk of inference in the record, indirect observation requires greater caution in data quality control (Anguera, 2020). Therefore, the concordance of the registry was measured by calculating the Cohen (1960), in the intra-observer mode, and yielded a value of 0.708, considered adequate in this study (Landis and Koch, 1977). In addition, consensus agreement (Lapresa et al., 2021) was used as a qualitative method of data quality control among three of the researchers in this study.

**Figure 2:**
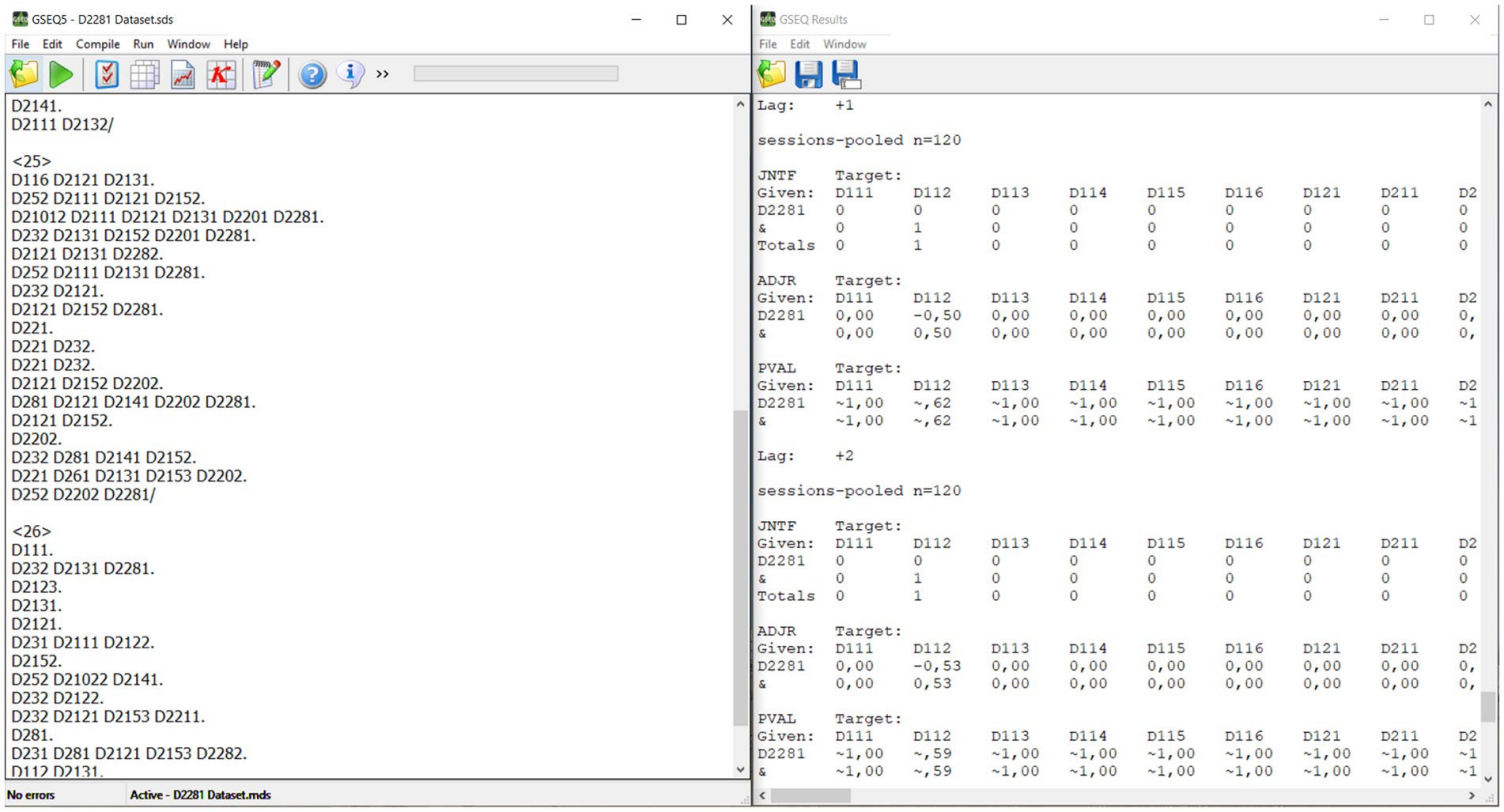
Matrix of codes obtained from the transcription of the interviews and lag sequential analysis with the GSEQ5 software. Reproduced with permission from Bakeman and Quera (2011).

Once the recorded interviews were transcribed, they were segmented into textual units under the spelling and syntactic criteria (Krippendorff, 2018). In total, the transcript of the interviews consisted of 73,046 words and 2,410 textual units. These units were recorded and coded by indirect observation, which involved carrying out the “quantitizing” operation (Anguera et al., 2020; Anguera, 2021), starting from the qualitative data of the interview to codify them thanks to the observation instrument, and thus obtaining a matrix of codes (Figure 2) that allowed a subsequent robust quantitative analysis.

### 2.6. Data analysis

Two types of analysis were carried out in this study, in order to detect the existence of regularities or communicative patterns, on the one hand, and to obtain a map of interrelationships between behaviors, on the other, during the communicative pattern established in the interviews.

First, a lag sequential analysis (LSA) (Bakeman, 1978) was performed to detect the possible existence of patterns of behavior among textual units obtained from interviews about the criteria that determine offensive success in elite women’s football. In this analysis, behavior *D2281: Linking success in offensive action or in the game in a positive way* was proposed as the target, which meant that it had the role of initializing the communicative patterns. The rest of the behaviors of the indirect instrument were considered conditioned (which implied knowing if they were part of the communicative patterns analyzed). This target behavior was selected because it represented those allusions made to offensive success in women’s football during interviews with coaches and players. Sequential analysis was conducted using the GSEQ software option to differentiate responses corresponding to each question as “units” to preserve intra-response sequentiality without affecting subsequent question responses. From this analysis, used satisfactorily in indirect observation (García-Fariña et al., 2018; Venturella et al., 2019; Del Giacco et al., 2020), the communicative pattern between target behaviors and conditioned behaviors was constructed. Although lags from −5 to +5 were analyzed, in this study, we focused on the results obtained in the −2 to +2, knowing that in higher lags the communicative pattern could become diluted (García-Fariña et al., 2018). Behaviors with a value greater than 1.96 obtained from the adjusted residual values obtained in this analysis were taken as significant (*p* < 0.05).

Second, to obtain a map of interrelationships between behaviors, a polar coordinate analysis (PCA) was carried out for which the target behavior of the sequential analysis was taken as focal, and the same conditioned behaviors. This analysis has been applied in other indirect observation studies in the field of sports (García-Fariña et al., 2018, 2021; Nunes et al., 2022) and other areas (Alcover et al., 2019; Arias-Pujol and Anguera, 2020; Del Giacco et al., 2020; Alvarado et al., 2021). For this analysis, which operates with the adjusted residual values obtained from the lag sequential analysis (LSA), lags −5 to +5 were taken into consideration (Anguera et al., 2018). The lengths and angles of each of the vectors (one for each of the conditioned behaviors) were calculated. These vectors indicate the type of relationship between the focal behavior and each conditioned behavior, and vice versa, as well as the intensity of such relationships. Based on these values, a vector map of the relationships between focal behavior and the rest of the conditioned behaviors was constructed.

The data has been exported using Lince Plus software into a matrix of codes^2^ (Soto-Fernández et al., 2021). All analyses were performed with the free program HOISAN (v 2.0)^3^ (Hernández-Mendo et al., 2012), with the exception of the calculation of lag 0, carried out using the free software GSEQ5^4^ (Bakeman and Quera, 2011).

## 3. Results

A total of 2,410 textual units were recorded and analyzed in the eight interviews. The behavior taken as target and focal for lag sequential analysis (LSA) and polar coordinate analysis (PCA) respectively was *D2281: Linking success in offensive action or in the game in a positive way*. The other behaviors of the indirect observation instrument were proposed as conditional for both types of analysis.

The results obtained from the lag sequential analysis reflected in Table 2 allowed us to verify how a total of 20 conditioned behaviors of the indirect observation instrument presented a statistically significant association from the value of the adjusted residual. Of the 20 behaviors, only 14 were taken into consideration, as they are related to the performance in women’s football. The behaviors were: D223: Refers to a specific player (Lag −2, *Z* = 2.22; Lag −1, *Z* = 2.63; Lag 0, *Z* = 4.00), D232: Refers to a combinative attack or position play (Lag −1, *Z* = 3.01; Lag 0, *Z* = 4.02), D261: Refers to the offensive transition (Lag 0, *Z* = 2.28), D271: Refers to set piece actions (Lag 0, *Z* = 3.16; Lag +1, *Z* = 2.49), D282: Refers to the defensive tactical intention of a team (Lag −1, *Z* = 4.06), D2912: Collective offensive technique (Lag 0, *Z* = 2.57), D21011: Individual offensive tactic (Lag −2, *Z* = 2.15), D2144: Refers to action space (Lag 0, *Z* = 2.68), D2153: Refers to the duration of the attack (Lag 0, *Z* = 2.00; Lag +1, *Z* = 2.57; Lag +2, *Z* = 2.11), D2161: Refers to the physical characteristics of a particular player (Lag−1, *Z* = 2.55), D2182: Refers to a dynamic startup type (Lag 0, *Z* = 6.39), D2191: Refers to an organized or structured defensive structuring (Lag −2, *Z* = 2.97), D2221: Refers to the number of passes that are made in an action (Lag −2, *Z* = 1.99), D2242: Refers to competition format (Lag +1, *Z* = 2.23; Lag +2, *Z* = 2.27).

**Table 2:** Lag sequential analysis results showing the relationship between criteria behavior (*D2281*) and the rest of the conditioned behaviors.

Lag −2	Lag −1	Lag 0	Lag +1	Lag +2
D223 (*Z* = 2.22) D21011 (*Z* = 2.15) D2191 (*Z* = 2.97) D2221 (*Z* = 1.99)	D223 (*Z* = 2.63) D232 (*Z* = 3.01) D282 (*Z* = 4.06) D2161 (*Z* = 2.55)	D211 (*Z* = 2.74) D223 (*Z* = 4.00) D232 (*Z* = 4.02) D261 (*Z* = 2.28) D271 (*Z* = 3.16) D2912 (*Z* = 2.57) D2144 (*Z* = 2.68) D2153 (*Z* = 2.00) D2182 (*Z* = 6.39)	D271 (*Z* = 2,49) D2153 (*Z* = 2,57) D2242 (*Z* = 2,23) D2271 (*Z* = 2,75)	D111 (*Z* = 2,01) D112 (*Z* = 2,59) D114 (*Z* = 2,25) D2153 (*Z* = 2,11) D2242 (*Z* = 2,27)

Results expressed as the value of the adjusted residual obtained for each of the behaviors analyzed.

**Figure 3:**
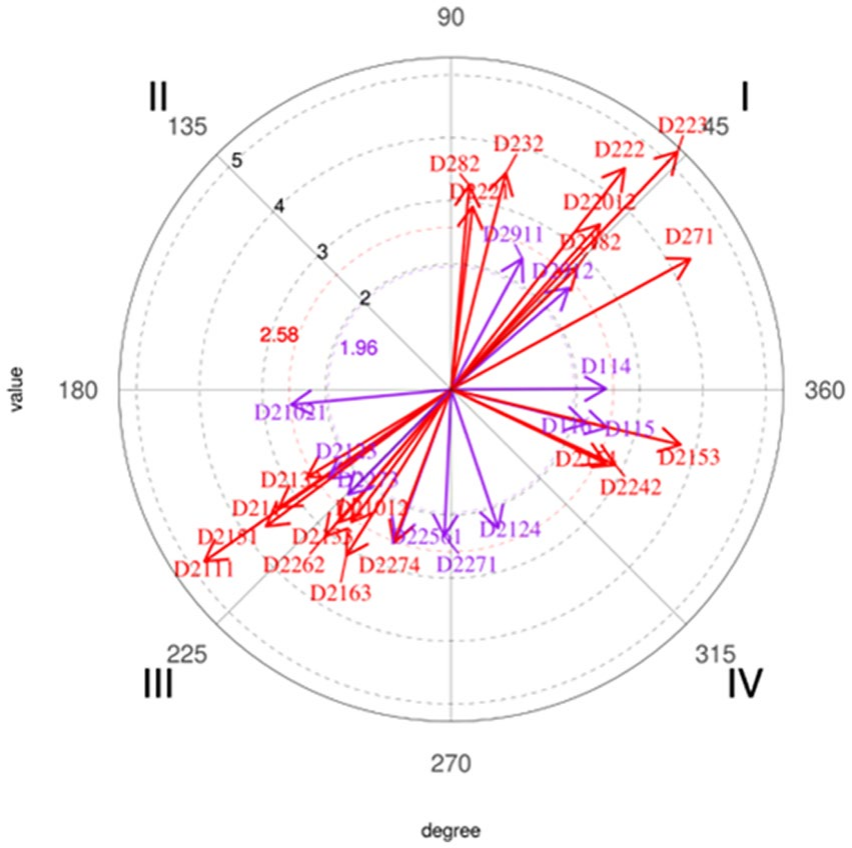
Polar coordinate analysis. Focal behavior D2281. Vector map.

Only those conditioned behaviors represented in quadrant I (prospective and retrospective activation with focal behavior) of the vector map with a radius equal to or greater than 1.96 were taken into consideration. Therefore, from the results obtained from the polar coordinates analysis (PCA) (Figure 3 and Table 3), a prospective and retrospective association of activation was found between the focal behavior D2281 and the behaviors considered as conditioned, represented in quadrant I of the vector map in eleven (11) conditioned behaviors analyzed in relation to the focal behavior. Of the 11 behaviors that were significant, 8 of them agreed with the results obtained from the lag sequential analysis. On the other hand, behaviors D222: Refers to a specific position, D2911: Individual offensive technique and D2201: Refers to decision making and perception, presented significant results for the objective of the study.

**Table 3:** Polar coordinate analysis results showing the relationship between focal behavior (*D2281*) and the rest of the conditioned behaviors.

Code	Quadrant	P. Prosp.	P. Retros.	Radius	Angle
D114	I	2.46	0.01	2.46*	0.31
D115	IV	2.48	−0.6	2.55	346.3
D116	IV	2.15	−0.55	2.22	345.77
D211	III	−2.74	−1.87	3.32	214.4
D222	I	2.75	3.52	4.47*	51.91
D223	I	3.6	3.78	5.22*	46.42
D232	I	0.87	3.44	3.55*	75.84
D271	I	3.8	2.08	4.33*	28.66
D282	I	0.29	3.25	3.26*	84.96
D2911	I	1.13	2.08	2.37*	61.55
D2912	I	1.87	1.61	2.47*	40.82
D21012	III	−1.59	−2.11	2.64	233.05
D21021	III	−2.55	−0.24	2.56	185.41
D2111	III	−3.92	−2.73	4.78	214.86
D2123	III	−1.95	−1.42	2.41	216.08
D2124	IV	0.75	−2.21	2.33	288.68
D2132	III	−2.29	−1.39	2.68	211.33
D2133	III	−1.79	−2.12	2.77	229.78
D2151	III	−2.95	−2.18	3.66	216.46
D2153	IV	3.65	−0.88	3.75	346.43
D2154	IV	2.46	−1.18	2.72	334.4
D2163	III	−1.66	−2.63	3.12	237.72
D2182	I	2.01	1,94	2.79*	44.03
D2201	I	2.36	2.63	3.53*	48.03
D2221	I	0.34	2.91	2.93*	83.33
D2242	IV	2.61	−1.2	2.87	335.31
D2251	III	−0.87	−2.39	2.55	249.97
D2262	III	−2	−2.33	3.08	229.36
D2271	III	−0.11	−2.33	2.33	267.36
D2273	III	−1.64	−1.67	2.34	225.62
D2274	III	−0.92	−2.44	2.61	249.42

P. Prosp, Prospective perspective; P. Retros, Retrospective perspective. *Significant behaviors (Radius > 1.96; *p* < 0.05) of prospective and retrospective activation with the focal behavior (Quadrant I).

The results obtained have made it possible to verify: (i) the existence of a large number of short patterns of behavior from the lag sequential analysis (LSA), as well as, through the application of the polar coordinate analysis (PCA) as a powerful data reduction technique: (ii) a map of interrelationships between behaviors established during the interviews carried out. Based on the interviews carried out, the criteria that, based on the opinions of the interviewees, condition the offensive success in women’s football are listed below. The following lines mention those behaviors extracted from the observation instrument that were significant in the analyses carried out, as well as examples extracted from the interviews carried out:

D223: Refers to a specific player (LSA: Lag −2, *Z* = 2.22; Lag −1, *Z* = 2.63; Lag 0, *Z* = 4.00 – PCA: Radius = 5.22, Angle: 46°42′):

Coach 1: *“Speed in women’s football is very decisive, Oshoala is a very fast player, if you leave space behind your back, you are dead.”*

Player 2: *“In that play the characteristics of the player (the power, the speed) make the action end successfully.”*

D232: Refers to a combinative attack or position play (LSA: Lag −1, *Z* = 3.01; Lag 0, *Z* = 4.02 PCA: Radius = 3.55, Angle: 75°84′):

Player 3: *“Successful teams have more ball possession, they keep the ball for more time, they have more passes in the attack. Everything causes physical wear to the unsuccessful team, which in the first part is not visible, but in the second it is.”*

Coach 4: *“It is assumed that in a positional attack you perceive how the rival team is positioned and you do it better than in a faster attack, with the disorganized rival team where you have to make decisions in less time.”*

D261: Refers to the offensive transition (LSA: Lag 0, *Z* = 2.28):

Player 3: *“In women’s football there are many transitions, many quick counterattacks […] and it is very successful.”*

Player 2: *“In my opinion […] in women’s football more goals are achieved in offensive transitions.”*

D271: Refers to set piece actions (LSA: Lag 0, *Z* = 3.16; Lag 1, *Z* = 2.49 – PCA: Radius = 4.33, Angle: 28°66′):

Coach 2: *“We could talk about set pieces as something different, because if we talk about success, normally a team that manages to score in set piece is usually associated with success in a match.”*

Player 3: *“There are many goals in corners, fouls*, etc. *It is very effective. If we do a study of the goals that are scored, there are many games in which you score in set piece.”*

D282: Refers to the defensive tactical intent of a team (LSA: Lag−1, *Z* = 4.06 – PCA: Radius = 3.26, Angle = 84°96′):

Player 1: *“I believe that it is a common trend that the best teams are able to: once they lose the ball perform a good pressure after loss and from there re-organize themselves to regain possession in the rival field.”*

Player 3: *“In the case of France it usually has more ball possession, it gets higher, so pressure after loss is its best strategy. […] USA is different because it can do the pressure after a loss and may not do it because it feels comfortable running behind it. Both are successful teams and pressure after loss is the strategy because they are the dominating teams […]. It does not make sense that being in a rival field 80 meters from your goal, you go back 50 meters.”*

Coach 5: *“I believe that the success, or one of the successes of a team that evidently pressures in the opposite field and steals after loss is that having the opposing team quite far from his goal, they steal near the opposite goal […]. Recovering the ball in that situation allows them to be more successful, be close to the opponent’s goal when he recovers […] and be more vertical.”*

D2912: Collective offensive technique (LSA: Lag 0, *Z* = 2.57 – ACP: Radius = 2.47, Angle = 40°82′):

Coach 1: *“To get a team that is defensively disorganized out of balance, if you are not able to have a good time, to pass it decisively, to know when it is on foot, when it is in space, to get that quality in the pass, it is impossible.”*

Player 1: *“I believe that having greater efficiency in the pass allows you to have greater control of the ball, more control over the opponent and in the end to be able to move them more so that the spaces are better.”*

Coach 4: *“Yes, of course, the key to the attack game is mainly the pass, either in direct game or in combinational game.”*

D21011: Individual offensive tactic (LSA: Lag −2, *Z* = 2.15):

Coach 3: *“The teams that are very superior at the end […] end up eliminating players for individual quality, they generate one on ones that end up solving and already the quality of the player generates qualitative superiorities. I think that’s where the advantage begins.”*

Player 3: *“What makes the differences is the technical and tactical quality of the players, so of course, if you have players who have a higher pass efficiency percentage, your conservation is much safer. Then your attack can be much longer if you decide, and you will be more effective.”*

D2144: Refers to action space (LSA: Lag 0, *Z* = 2.68).

Coach 5: *“I think that when a player does not have the ball, what interests me is what I provoke without having the ball: how my position makes the other players, or my companions, can play better, with more time, with more space. How, for example, my extremes and my tip are able to lengthen the rival team to generate spaces to our interiors, to our mid-centers and that is done, obviously with breadth and depth. If we want to have a game of position inside, the position of the far ones in amplitude and depth is fundamental.”*

Player 3: *“I think the most talented players make the difference because they do not need that space and that time: their decision making is much faster than the rest, their technical quality makes them not need that space because they are going to leave control here, and not there.”*

D2153: Refers to the duration of the attack (LSA: Lag 0, *Z* = 2.00; Lag +1, *Z* = 2.57; Lag +2, *Z* = 2.11 – PCA: Radius = 3.75, Angle = 346°43′):

Player 3: *“I can give you the example of USA which I think is a team that feels like defending in its own field and coming out in fast attack, with short possessions […] The current world champion was USA and in the Canadian World Cup it was USA.”*

Player 2: *“I think more goals are achieved in offensive transitions.”*

D2161: Refers to the physical characteristics of a particular player (LSA: Lag −1, *Z* = 2.55).

Coach 1: *“In elite football, the players who make the difference are the fastest players; they are usually the ones that cause more unbalance on opponents, the most skilled players. That in women’s football does not exist so much because of what we have said before: that speed does not exist, in fact, there are 3–4 very fast players and they call attention powerfully because they are very different from the others and they are the ones that make the difference.”*

Coach 5: *“In the specific case of Nigeria that has a player like Oshoala that allows you this, well,… nowadays there are more and more players with more power at that level, with more capacity. For example Poland has Pajor who is another player who has a different speed than the rest and allows her team to play that way. Are there more and more styles in women’s football in which we can see that the transition is decisive? I think so. Because there are more and more players of this style […] Then it depends a little on the individual characteristics of this type of player. Whether or not you have one on your team allows you to play that way or at some point opt for this game idea.”*

D2182: Refers to a dynamic startup type (LSA: Lag 0, *Z* = 6.39 – PCA: Radius = 2.79, Angle = 44°03′):

Player 3: *“I understand that it is more advantageous in dynamic because there is more disorder.”*

Coach 5: *“I think that after gaining possession it can be more advantageous, because after stealing possession you get the rival team to be a little more disorganized and if you attack it well it is possible that you can have more advantage in that sense.”*

Player 1: *“In the end I believe that the dynamic way is the most advantageous […] Because in the end when you are inside the game the positions are not exact. If you manage to steal the ball, make an interception or appropriation to the team you steal from, it will not be well positioned defensively at that moment.”*

D2191: Refers to an organized or structured defensive structuring (LSA: Lag −2, *Z* = 2.97).

Coach 3: *“Static, so to speak, gives us more time for prior organization, and dynamic does not give us time for that prior organization. In the second, we must be faster in organizing ourselves well and a key factor that is very important for me in everything dynamic, which is once again the technical execution and the decision-making capacity. That I think that in feminine football is worse and therefore I think there would be more advantage in static.”*

Coach 4: *“In static you can see the options you have to attack, you can perceive them better because it is assumed that in positional attack you contemplate how the rival team is positioned and you can perceive better than perhaps in a faster attack with the disorganized rival team where you have to make the decisions in less time, because this way, it is assumed that you have more time to perceive and decide.”*

D2221: Refers to the number of passes that are made in an action (LSA: Lag −2, *Z* = 1.99 – PCA: Radius = 2.93, Angle = 83°33′):

Coach 2: *“In the end the more possession you have in the rival field is better. I think that ball possession in our side of the field is sterile and there are also different studies that reaffirm it; you are not more successful for making more passes in your own side of the field but for making more passes in the opponent’s field.”*

Coach 3: *“Obviously giving more passes the opponent has to do other things (it has to be adjusted) and if you execute this number of passes well and you are able to turn the opponent, to build an attack passing through different areas, you take to the side to generate “there,” you make the opponent close distance between its members to generate in an area,… If all that is well developed I think you can generate certain imbalances that you can then take advantage of.”*

Coach 5: *“I believe that a team that bases its attack on the combinational game to perform a greater number of passes will allow it to disorganize the opponent more.”*

D2242: Refers to competition format (LSA: Lag +1, *Z* = 2.23; Lag +2, *Z* = 2.27 – PCA: Radius = 2.87, Angle = 335°31′):

Coach 1: *“I think a fundamental aspect of the difference between playing a league and a championship at the end is that you are playing it in a match. So in that game you know that if you put yourself ahead on the scoreboard, you win it, you pass the elimination round and the opposing team is out therefore from that moment you seek to consolidate that put yourself ahead on the scoreboard. This is the moment to be more defensively solid, to maintain more possession of the ball, to try to maintain more possession of the ball without risking it perhaps so much and to propitiate the desperation of the rival and that this desperation of the rival propitiates you to be able to harm him in moments of imbalance. That the opposing team in that wanting to steal possession and in that anxiety for wanting to neutralize the adverse scoreboard can be disorganized and that you can waste it.”*

Player 1: *“If you feel very comfortable playing the game and you advance on the scoreboard you can continue to maintain your level of play and you do not need to modify much more unless the opponent goes much more on the attack and you look much more enclosed. Then you have to change your game strategy, you will have to keep the result a little longer by giving the possession to the opponent and trying to find more balls against them. So I think that the factor of how the team is at that time is very important, of whether it is comfortable or not, and especially the minute that it is and that it is a single game, since that conditions you to have to preserve the result much more than if it were a league mode.”*

D222: Refers to specific position (PCA: Radius = 4.47, Angle = 51°91′):

Coach 2: *“Many teams in the lower part had a pair of powerful and fast forwards that after stealing possession were only sought after and that they had to manage themselves.”*

Player 3: *“If we think about the style of play that has USA (which is a transition team) and it really has very fast players, very strong against, I think that in that sense, at least so far many of the teams that won or that were successful teams in the female category was because they made a difference on a physical level.”*

D2911: Individual offensive technique (PCA: Radius = 2.37, Radius = 61°55′):

Player 1: *“In the end successful selections are successful because they have better players, so possession is obviously better because there is a different technical-tactical level in those players. I believe that when you have good players and a higher tactical level, because in the end they are players who are in good teams and in good categories, and in the end that makes the team tidy up better tactically. If you have a good tactical disposition and your players are technically better, the possession will be better and you will have more guarantees that the possession will be successful.”*

Player 3: *“It is clear that in the end when your players are better in the pass, technically and tactically they are better, that gives you confidence […] and your game model is going to be that.”*

D2201: Refers to decision making and perception (PCA: Radius = 3.53, Angle = 48°03′):

Coach 2: *“Football is constant perception and decision making. The less space you have, the less time you have to perceive and make decisions. […] Many times we talk about taking away your space, when really what they are taking away is the time for that decision making. What does that force you to do? To “that thinking before.” In such situations we always remember footballers like Xavi Hernández who was always looking everywhere before receiving the ball, so when he received it he already had many more options thought out, many more answers; that’s what really gave speed to the game.”*

## 4. Discussion

This study was carried out with the aim of detecting regularities in the offensive success in elite women’s football, as well as carrying out an interrelational analysis of linked behaviors, based on in-depth interviews with professional coaches and players. Along these lines, different behaviors linked to offensive success in women’s football have been found in the communicative pattern of the interviews carried out.

This study’s coaches and players suggested a link between attack type (D232: Refers to a combinative attack or position play – LSA: Lag −1, *Z* = 3.01; Lag 0, *Z* = 4.02 PCA: Radius = 3.55, Angle: 75°84′) and successful possessions in women’s football, such as entering the rival area or shooting on goal. In the same way, previous studies confirmed this influence. For example, Scanlan et al. (2020) found that possessions initiated by an interception were more likely to end on score-box possessions at the FIFA Women’s World Cup (FWWC) 2015. Similarly, at the FWWC France 2019, possessions that started near the rival goal to advance quickly also reached the rival goal more often (Iván-Baragaño et al., 2021), confirming these findings with the link made in the communicative pattern of the interviewees between offensive transition and success in the attack (D261: Refers to the offensive transition – LSA: Lag 0, *Z* = 2.28). These findings agree with existing literature and could imply that fast attacks, specifically counterattacks, are more effective than positional attacks in women’s football. In fact, activation patterns were found in indicators such as the duration of the attack (D2153: Refers to the duration of the attack – LSA: Lag 0, *Z* = 2.00; Lag +1, *Z* = 2.57; Lag +2, *Z* = 2.11 – PCA: Radius = 3.75, Angle = 346°43′) and the dynamic startup type (D2182: Refers to a dynamic startup type – LSA: Lag 0, *Z* = 6.39 – PCA: Radius = 2.79, Angle = 44°03′) which were directly linked to the playing style of the most successful women’s football team as follows: “*USA is a team that feels like defending in its own field and coming out in fast attack, with short possessions […] The current world champion was USA and in the Canadian World Cup it was USA.”* In addition, the interviewees mentioned a common but often neglected aspect of football matches: set pieces (D271: Refers to set piece actions LSA: Lag 0, *Z* = 3.16; Lag 1, *Z* = 2.49 – PCA: Radius = 4.33, Angle: 28°66´). As one interviewee said, “if we talk about success, usually a team that scores in set piece is associated with success in a match.” Lee and Mills (2021) showed that different variables (e.g., type of ball delivery or the number of players involved) affected the probability of completing these actions through analysis at the FWWC France 2019. Therefore, the interviewees’ perceptions seem to agree with the scientific evidence.

On the other hand, responses to interviews suggested a relationship between a player’s physical characteristics (D2161: Refers to the physical characteristics of a particular player LSA: Lag −1, *Z* = 2.55) and offensive success. In this line, the lack of scientific evidence does not facilitate the extrapolation of these subjective impressions to the practical field. Nevertheless, some data collected by the official FIFA report on FWWC France 2019 (FIFA, 2019c) seem to indicate that this relationship may exist taking into account that the teams that managed to reach the semi-final round were able to run more meters at high intensity and perform a greater number of sprints per match. Based on these results, we could consider that conditional training is playing (and will play) a fundamental role in the performance of teams in the most important women’s football championships. In relation to the individual and collective technical elements, the results of this study (D2912: Collective offensive technique (LSA: Lag 0, *Z* = 2.57) – ACP: Radius = 2.47, Angle = 40°82′); D21011: Individual offensive tactic (LSA: Lag −2, *Z* = 2.15) seem to agree with previous results in which differences in the time of possession in the rival field were evidenced (Iván-Baragaño et al., 2022). In fact, there have been several studies that have analyzed these technical variables and, in particular, the differences between men’s football and women’s football (Bradley et al., 2014; Casal et al., 2021; Garnica-Caparrós and Memmert, 2021; Pappalardo et al., 2021). In this sense, it could be considered that the improvement of individual technical performance would be a condition that would increase performance and, consequently, the probability of successfully completing offensive actions in women’s football.

Finally, reference is made to an aspect discussed during the interviews with the players: the speed of decision-making D2201: Refers to decision-making and perception (PCA: Radius = 3.53, Angle = 48°03′). While it is true that on this subject no research allows us to contrast these opinions strongly, we can agree that the greater the speed in decision-making, the greater the speed in the game and the easier it is to defensively mess up the rival team. In fact, this speed in the game has been compared by Pappalardo et al. (2021) between men’s football and women’s football, being greater in the case of male players, giving a margin for improvement in the case of women’s football and allowing, following the interviewees, to increase the probability of offensive success in women’s football teams.

## 5. Conclusion

In this study, a large number of indicators that can determine offensive success in elite women’s football were suggested. Using a novel technique and not often used in the field of sport, significant associations have been found established in the communicative pattern that emerged during the in-depth interviews carried out. Specifically, success in women’s football was associated with (i) the conditional characteristics of a player, (ii) offensive transition and the type of dynamic start to ball possession, (iii) set-piece actions, (iv) collective defensive intention, specifically pressure after loss, (v) collective technical performance, (vi) the number of passes, or (vii) decision-making. Many of the indicators mentioned in this study as possible performance indicators in women’s football have already been studied in men’s football. Despite this, it is still unknown whether the results extracted from studies carried out with a male sample can be extrapolated to the opposite sex. Therefore, the study of future researchers focused on the analysis of match performance in women’s football may be oriented toward demonstrating the influence of these criteria on performance in competition.

## 6. Futures lines of research

In the near future, women’s football researchers may propose studies that try to know the influence of the dimensions suggested in this study on success in women’s football. In this sense, study aspects such as: (i) the influence of the physical performance of a player or a specific position on the success of a team or selection; (ii) the degree of offensive effectiveness achieved depending on the type of attack used; (iii) the importance of set pieces as decisive actions for the final result of the match; (iv) the degree of association between individual and collective technical-tactical performance and the degree of success achieved by a team in a match or championship; (v) the influence of the competition format on the offensive behavior of the teams; (vi) the modification of the probability of obtaining goal scoring opportunities in the possessions of the ball depending on the number of passes, the type of start, or the defensive tactical intention or; and (vii) the association between the speed of decision-making and the offensive and defensive success of a team or selection will increase the scientific corpus on this subject and, inevitably, increase the performance in matches of teams and selections in elite women’s football.

On the other hand, it is seen as crucial that ongoing research expands the understanding and perspectives of specialists in women’s football. In this vein, conducting research employing the Delphi Method with coaches and players from various nations might aid in advancing scientific understanding of the most crucial performance indicators in women’s soccer.

## Data availability statement

The raw data supporting the conclusions of this article will be made available by the authors, without undue reservation.

## Author contributions

II-B, MTA, RM, and AA: research concept and study design. II-B, RM, JL, and MTA: literature review and writing of the manuscript—original draft preparation. II-B, MTA, and JL: conceptualization, methodology, data analysis and interpretation, and statistical analysis. MTA, RM, and JL: formal analysis, investigation, and resources. II-B, RM, and AA: data collection. II-B, AA, MTA, JL, and RM: writing—review and editing. All authors have read and agreed to the published version of the manuscript.

## Conflict of interest

The authors declare that the research was conducted in the absence of any commercial or financial relationships that could be construed as a potential conflict of interest.

## Publisher’s note

All claims expressed in this article are solely those of the authors and do not necessarily represent those of their affiliated organizations, or those of the publisher, the editors and the reviewers. Any product that may be evaluated in this article, or claim that may be made by its manufacturer, is not guaranteed or endorsed by the publisher.

## Footnotes

^1^ https://observesport.github.io/lince-plus

^2^ https://observesport.github.io/lince-plus

^3^ www.menpas.com

^4^ https://www.mangold-international.com/en/products/software/gseq.html
